# A Comparison of Antegrade Percutaneous and Laparoscopic Approaches in the Treatment of Proximal Ureteral Stones

**DOI:** 10.1155/2014/691946

**Published:** 2014-09-11

**Authors:** Hikmet Topaloglu, Nihat Karakoyunlu, Sercan Sari, Hakki Ugur Ozok, Levent Sagnak, Hamit Ersoy

**Affiliations:** ^1^Urology Clinic, Diskapi Yildirim Beyazit Training and Research Hospital, 06310 Ankara, Turkey; ^2^Department of Urology, Hitit University Faculty of Medicine, 19030 Corum, Turkey

## Abstract

*Purpose*. To compare the effectiveness and safety of retroperitoneal laparoscopic ureterolithotomy (RLU) and percutaneous antegrade ureteroscopy (PAU) in which we use semirigid ureteroscopy in the treatment of proximal ureteral stones. *Methods*. Fifty-eight patients with large, impacted stones who had a history of failed shock wave lithotripsy (SWL) and, retrograde ureterorenoscopy (URS) were included in the study between April 2007 and April 2014. Thirty-seven PAU and twenty-one RLU procedures were applied. Stone-free rates, operation times, duration of hospital stay, and follow-up duration were analyzed. *Results*. Overall stone-free rate was 100% for both groups. There was no significant difference between both groups with respect to postoperative duration of hospital stay and urinary leakage of more than 2 days. PAU group had a greater amount of blood loss (mean hemoglobin drops for PAU group and RLU group were 1.6 ± 1.1 g/dL versus 0.5 ± 0.3 g/dL, resp.; *P* = 0.022). RLU group had longer operation time (for PAU group and RLU group 80.1 ± 44.6 min versus 102.1 ± 45.5 min, resp.; *P* = 0.039). *Conclusions*. Both PAU and RLU appear to be comparable in the treatment of proximal ureteral stones when the history is notable for a failed retrograde approach or SWL. The decision should be based on surgical expertise and availability of surgical equipment.

## 1. Introduction

Current treatment of proximal ureteral stones poses a challenge especially in large and impacted stones, or when SWL and retrograde ureteroscopy fail. SWL and retrograde ureterolithotomy are current treatment methods in the management of proximal ureteral stones because of their minimally invasive characters. Recently, the flexible ureteroscopes have become the main actors in retrograde ureterolithotomy. However, semirigid ureteroscopy is still one of the major actors in developing countries due to the lack of technological facilities.

Percutaneous antegrade extraction of ureteral stones may be considered in some selected cases including very large (diameter ≥ 15 mm) impacted stones located at proximal ureter or between ureteropelvic junction and lower border of the 4th lumbar vertebra [1, 2]. In addition, it may be considered as an option when SWL is not indicated or failed [3] or when upper urinary system cannot be accessed with retrograde ureteroscopy.

Laparoscopic ureterolithotomy is another good alternative to open surgical technique in very large, impacted, and/or multiple ureteral stones when URS and SWL fail and the surgeon is experienced in laparoscopic techniques. Both retroperitoneal and transperitoneal interventions have been reported for each segment of ureter [4]. Although both laparoscopic ureterolithotomy and PAU are highly effective, they are associated with longer operation times, duration of hospital stay, time to return to normal activity, and increased complication rates compared to SWL and URS [2, 4].

The aim of this study was to compare the effectiveness and safety of RLU and PAU with semirigid ureteroscopy in the treatment of proximal ureteral stones.

## 2. Material and Methods

This retrospective study included 58 patients who had a history of failed SWL or retrograde ureteroscopy or were diagnosed to have a large (diameter ≥ 15 mm) impacted proximal ureteral stone between April 2007 and April 2014 (Table 1). These methods may be considered as first-line therapy in large impacted stones with severe hydronephrosis (≥grade 2) when a delay related to SWL failure is not tolerable. PAU group did not include patients with ureteropelvic stones concurrent with renal stones. The motive of this exclusion was the concern of forming a homogeneous group with respect to duration and side effects for comparison with RLU group. RLU group did not include patients with previous laparoscopic interventions with the exception of prior retroperitoneal open surgery or treatment for ureteral stone. Indications for PAU and RLU were approved by the council of authors. While the PAU method was preferred for stones close to the kidney or the presence of dilated ureter due to obstruction due to the stone, RLU was selected for small stones or stones close to L4 level that did not cause dilatation.

*Surgical Techniques.* In the PAU procedure, all patients underwent general anesthesia and a 6 F open-ended ureter catheter was first placed via transurethral approach. Percutaneous access was performed under fluoroscopy guidance with the patient in the prone position using 18 G access needle, and the middle calyx was preferentially used. A 0.89 mm J-tipped guide wire was placed in the collecting system, and skin and fascia were incised before the access needle was retracted. The nephrostomy tract was dilated by using Alken metal dilators (Karl Storz GmbH, Tutlingen, Germany) or serial polyurethane Amplatz dilators (Mikrovasive, Natick, MA, USA). A 30 F Amplatz sheath (Mikrovasive) was inserted by aiming at renal collecting system. A semirigid ureteroscope (Olympus) was used to access the ureteral stone. The stones were fractionated using a pneumatic lithotripter. Following confirmation of complete clearing of stones both endoscopically and fluoroscopically, reentry pigtail catheter (Mikrovasive) was placed antegradely [5].

In the RLU procedure, 3 (10–12 mm) trocars were used. The first port was placed via the open method at the junction of the lower edge of the 12th rib and posterior axillary line. In the open method, a 1.5 cm incision was performed and continued under direct vision to the fascia of the external oblique muscle. Fascia of the transversus abdominis muscle was punctured with a blunt clamp and retroperitoneal space was accessed. An 800 cc space was created first with a finger and then with a balloon dissector while the peritoneum was shifted at the anterior face of the Gerato fascia at the same time. The second port was placed 1 cm anterior to the 11th rib. The third port was placed at the level of the anterior axillary line, 2 cm superior and 2 cm medial to spina iliaca anterior superior. After the area was widened, the Gerato fascia was opened, and the ureter was accessed over the psoas muscle. The protuberance of the stone was palpated and caught with a Babcock grasping clamp from the side close to the renal pelvis. Following stabilization of the stone, the ureter was opened vertically using a wedge-tipped endoscopic scalpel. The stone was extracted with a right-angle forceps. It was placed in an endobag, and a 25 cm double J (DJ) ureteral catheter was inserted endoscopically. The ureteral incision was closed intracorporeally using a 4/0 Vicryl suture. A hemovac drainage catheter was placed in the periureteric area where the 2nd port was placed. The DJ catheter was left in place for 5 days. The drains were then removed upon a decrease of the amount of fluid drained to less than 50 cc daily [6].

Demographic features of the patients, as well as stone characteristics, stone-free rates, duration of urinary leakage, operation times, mean drop in hemoglobin levels, duration of hospital stay, and follow-up, were analyzed for each group. The surface area of the opaque stones was measured two-dimensionally by superimposing radiograms on a graphic paper. Nonopaque stones were measured with CT imaging. Clearance of stones was assessed with a kidney ureter bladder (KUB) graph taken at first postoperative day for opaque stones and with a CT examination taken at the first month for nonopaque stones. The definition of the duration of urinary leakage was the time between the end of the operation and cessation of the leak. Hemoglobin levels were studied as part of complete blood count one day before and after the operation.

The statistical analyses were carried out using “SPSS 11.5 for Windows” software package. Mann Whitney *U* test was used for the evaluation of intergroup differences of operation time, duration of urinary leakage, mean duration of hospital stay, duration of follow-up, and surface area of the stone. Differences between the groups with respect to re- and postoperative hemoglobin concentrations were analyzed using Student's *t*-test. Pearson's Chi-Square test was used for the comparison of differences regarding sex, side of the stone, clearance of stones, and fever >38°C. A *P* value less than 0.05 was considered statistically significant.

## 3. Results

Both groups were comparable due to similar demographics (Table 2). Overall stone-free rate was 100% for both groups. No significant differences were observed between the groups with respect to postoperative duration of hospital stay and urinary leakage more than 2 days. While the blood loss was significantly higher in PAU group (−1.6 ± 1.1 g/dL versus −0.5 ± 0.3 g/dL, *P* = 0.022), the mean operative time was significantly longer in RLU group (80.1 ± 44.6 min versus 102.1 ± 45.5 min, *P* = 0.039) (Table 2). Although our operation table was C-arm compatible, we did not need to use fluoroscopy for determination of stone localization in RLU group.

## 4. Discussion

The best treatment modality for large proximal ureteral stones is still debated. The available methods include medical expulsive therapy, SWL, ureterolithotripsy using semirigid or flexible tools and pneumatic or laser lithotriptor, PAU, laparoscopic ureterolithotomy, and open surgery [7]. Current literature data suggest variable success rates [7, 8]. The aim of treatment should be to provide freedom of stones as soon and as safely as possible and with the least invasive procedure possible. Determination of the best technique requires stone characteristics, anatomical details, patient status, and surgeon's preferences.

Selecting the best method for the treatment of stones should favor not only the least invasive procedure but also the most effective method. There is no doubt that SWL is the least invasive and laparoscopic ureterolithotomy is the most invasive method. However, despite its less invasive nature, SWL has some limitations in impacted stones. These limiting factors that preclude higher success rates include localization difficulties during SWL and lack of adequate space for stones to dilate during fragmentation [9]. Despite its effectiveness SWL, clearance of stones is usually injured by chronic inflammatory reaction related to polyps and inflammation associated with inflammation [10–12]. Ureteroscopic methods, on the other hand, have limited success especially in men due partly to difficulties in accessing stones, but mostly secondary to common ureteral lesions associated with impacted stones, such as edema, polyps, and strictures [13]. In such cases, a long history of impacted stones has been considered as a predictor [10]. In our study, the rate of unsuccessful SWL, URS, and SWL + URS was 77% (27/37 patients) in the PAU group and 76.2% (16/21 patients) in the RLU group.

Percutaneous transrenal insertion of ureteroscope is an effective means of accessing large or impacted stones especially in the proximal ureter. Relative advantages of PAU versus retrograde URS are as follows: opportunity for a more correct access to kidney (retrograde advancement of the guide wire is sometimes impossible with severely impacted stones), ability to use instruments of a larger size (a flexible cystoscope is usually used when hydroureteronephrosis is present above the stone), and ability to wash stone fragments towards the urinary bladder (instead of picking up stone fragments one by one or waiting for them to fall). Stone-free status is easily achieved. An additional invasiveness may be mentioned for PAU in primary use in case of very large and impacted ureteral stones. Vijay Kumar et al. [1] reported a procedural success rate of 86% in 86 patients with impacted ureteral stones. Despite the high-risk nature of this group, the perforation rate reported was only 9%. Maheshwari et al. [14] reported a success rate of 100% as we did and experienced no complications in 23 proximal ureteral stones, all of which were impacted and greater than 1.5 cm. Goel et al. [15] reported a complete stone-free status in 65 of 66 patients with impacted proximal ureteral stones larger than 1.5 cm.

Spectrum of complications with PAU, as expected, shows some variations compared with retrograde URS, depending on the retrograde entry to kidney. Three series including 175 PAU cases, which reported a 3% rate of hemorrhage requiring transfusion are summarized below [1, 14, 15]. In our series, hemoglobin drop in the PAU group was significantly higher (*P* = 0.022) than that of the RLU group though we did not detect any transfusion-requiring hemorrhage. In addition, increased invasiveness resulted from accompanying risk of fever development (15%) and general complications (14%). In conclusion, in highly selected cases with large or impacted stones who undergo this procedure, an injury rate of 5% and a ureteral stricture rate of 3% are expected compared to retrograde URS; however, we did not encounter such complications in our PAU group during a follow-up of a mean of 21.33 months though we also used semirigid ureteroscope.

The American Urological Association Ureteral Stones Clinical Guidelines (1997) stated that “open surgery should no longer be considered as a first-line treatment” for all stones at any localization of ureter [16]. This rule has naturally not been changed; however, a few small case series about laparoscopic ureterolithotomy have been published since the publication of this document. Some small, comparative series have argued that laparoscopic ureterolithotomy may replace open surgical ureterolithotomy in many circumstances [17]. Steps of the laparoscopic approach are similar to open surgery with the exceptions of entry to ureter and the instruments used. The overall success rate is 96%. Although stone size does not appear to be very significant in achieving the expected success, laparoscopic ureterolithotomy is less successful in the distal ureter than middle and proximal ureters.

Laparoscopic ureterolithotomy can be applied both retroperitoneally and transretroperitoneally. Both methods are equally effective, but the first technique is associated with a shorter recovery period [6, 18, 19]. RLU, compared to transperitoneal approach, has some advantages with respect to sparing of peritoneum and mobilization of internal organs. It also protects peritoneal space from urinary contamination. Furthermore, working area is limited in retroperitoneal approach and localizing ureter poses a challenge owing to scarceness of anatomical marking points. Indeed, Harewood et al. [20] reported a need for conversion from retroperitoneal approach to transperitoneal laparoscopy in 2 out of every 3 cases owing to inadequate surgical space. Hemal et al. [21] reported the lowest success rate ever (75%), which they attributed to the need of conversion to an open procedure at the beginning of the surgery as a result of the learning curve. In addition, Rassweiler et al. [22] reported 5 RLU cases among 200 retroperitoneal cases and added that this procedure should be improved in the future. To our opinion, the retroperitoneal approach complies with the surgical principles in upper urinary system approach. Moreover, as a technique, it is safe, simple, reproducible, and effective. Most complications are minor which are readily manageable.

Laparoscopic ureterolithotomy is a more invasive procedure than PAU with an overall complication rate of 21% [17]. The most common complication is prolonged urinary leakage (12%) that is usually defined as a urinoma formation or persistent urine discharge from the periureteral drain for more than 2 to 4 days. Despite the fact that most series have used periureteral drains, some others have not used drain, stent, or either. Stents and drains were recommended in 1/8 of prolonged urinary leakages. Wound site and urinary infections are more common, at a rate of 7%, than other procedures. The major complication of laparoscopic ureterolithotomy is stricture formation that has been reported to have a rate of 15–20% in different series [23, 24]. Nevertheless, that possibility has appeared at a rate of 2.5% in a work by Nouira et al. [25] where they summarized literature reviews. We did not observe any complication as strictures at a mean follow-up duration of 21.14 months. The etiology of postoperative ureteral strictures is unclear. Keeley et al. [23] considered that strictures that developed in their 2 patients were related to suturing during ureterotomy. Nouira et al. [25], on the other hand, suggested that too tight sutures cause ureteral strictures by creating ischemia. They argue that the suturing method should at all time aim to approximate ureter ends to facilitate healing, and they should not purport water resistance. Nouira et al. [25] reported that an incision made with a cold knife is more widely accepted since it offers a better wound healing and fewer strictures, whereas we feel that, as in the large series of Gaur et al. [26], ureter incision with an electric hook in cutting mode will be easier. Harewood et al. [20] used diathermy hook electrode for opening ureters in 6 patients and observed no ureter stricture. Mitchinson and Bird [27] suggested that prolonged urinary leakage concurrent with retroperitoneal fibrosis might be the possible cause of ureteral strictures.

Treatment of proximal ureteral stones involves multiple procedures until stone eradication is completed. Although RLU is associated with a higher success rate and a need of a lower number of interventions, it leads to more postoperative pain, longer procedure times, and prolonged durations of hospital stay, albeit statistically nonsignificant. While the role of RLU in the treatment is limited to being a second-step salvage surgery, it offers more advantages than open ureterolithotomy.

In our study, the overall stone-free rate was 100% for both groups. No significant difference was observed across groups with respect to postoperative duration of hospital stay and urinary leakage longer than 2 days. While blood loss was greater in the PAU group (*P* = 0.022), the operation time was longer in the RLU group (*P* = 0.039). The most important limitations of our study were the retrospective nature and the low number of study subjects. This was because the cases in whom an indication of PAU or RLU was to be put were canalized to RIRS with the introduction of flexible renoscopy at our clinic in February 2012. We believe that our study will bring a new perspective to the existing literature with regard to urology clinics being devoid of flexible URS.

## 5. Conclusions

In conclusion, in the treatment of proximal ureteral stones, both PAU and RLU offer suitable and comparable options when there is a history of unsuccessful retrograde manipulation or SWL and no flexible URS equipment is present. Surgical expertise and adequateness of available equipment will guide the selection.

## Figures and Tables

**Table 1:** Indication for treatment.

	PAU∗*n* (%)	RLU∗∗*n* (%)
Large, impacted ureteral stones with severe hydronephrosis	10 (27%)	5 (23.8%)
Failed SWL	5 (13.5%)	4 (19%)
Failed URS	4 (10.8%)	2 (9.5%)
Failed SWL + URS	18 (48.7%)	10 (47.7%)

Total	37 (100%)	21 (100%)

∗Percutaneous antegrade ureterolithotripsy.

∗∗Retroperitoneal laparoscopic ureterolithotomy.

**Table 2:** Comparison of two groups: the demographic and clinical data and the outcomes during and after surgery of 58 cases.

	PAU group	RLU group	*P*
	*n*: 37	*n*: 21	
Median age, years (range)	48 (17–77)	49 (20–79)	0.298
Female/male, *n* (%)	15 (40.6)/22 (59.4)	8 (38.1)/13 (61.9)	0.839
Stone side, right/left, *n* (%)	14 (37.8)/23 (62.2)	9 (42.8)/12 (57.2)	0.745
Stone surface area, mm^2^ (mean ± std.)	105.3 ± 77.6	117.4 ± 83.4	0.497
Decrease in haemoglobin, g/dL (mean ± std.)	−1.6 ± 1.1	−0.5 ± 0.3	0.022
Stone-free status, *n* (%)	37 (100)	21 (100)	1.000
Operative time, min. (mean ± std.)	80.1 ± 44.6	102.1 ± 45.5	0.039
Fever >38°C, *n*	4	2	0.801
Median hospital stay, days (range)	3 (2–6)	4 (2–7)	0.127
Median urine leakage time, days (range)	2 (1–5)	3 (1–6)	0.198
Follow-up (month) (mean ± std.)	21.33 ± 4.66	21.14 ± 5.49	0.959
